# Detection of Potential Zoonotic Agents Isolated in Italian Shelters and the Assessment of Animal Welfare Correlation with Antimicrobial Resistance in *Escherichia coli* Strains

**DOI:** 10.3390/antibiotics12050863

**Published:** 2023-05-06

**Authors:** Antonio Cocco, Alessandra Alessiani, Romolo Salini, Federica Iapaolo, Daniela Averaimo, Cinzia Pompilii, Giovanni Foschi, Fabio Bellucci, Filomena Iannino, Paolo Dalla Villa, Anna Janowicz, Marco Caporale

**Affiliations:** 1 Istituto Zooprofilattico Sperimentale dell’Abruzzo e Molise “G. Caporale”, 64100 Teramo, Italy; a.cocco@izs.it (A.C.); alessandra.alessiani@izspb.it (A.A.); r.salini@izs.it (R.S.); f.iapaolo@izs.it (F.I.); g.foschi@izs.it (G.F.);; 2 Istituto Zooprofilattico Sperimentale della Puglia e della Basilicata, 71121 Foggia, Italy; 3 Ministero della Salute, Direzione Generale della Sanità e dei Farmaci Veterinari, 00144 Roma, Italy

**Keywords:** shelters in Italy, animal welfare, zoonotic pathogens, AMR, SQP

## Abstract

Welfare conditions in shelters, where dogs might be housed for a long period of time, may have a possible correlation with the occurrence of bacterial pathogens and their antimicrobial resistance (AMR). In this study, we assessed the occurrence of AMR in 54 strains of *Escherichia coli* isolated from dogs housed in 15 Italian shelters and we correlated the resistance patterns to animal welfare. We also aimed to evaluate the presence of specific pathogens with zoonotic potential in sheltered dogs. Thus, nasopharyngeal, rectal, and oral swabs were collected from a group of 20 dogs in each shelter and totaled 758 swabs. We identified 9 *Staphylococcus pseudointermedius*, 1 *Pasteurella multocida*, 9 *Staphylococcus aureus*, 12 *Campylobacter* spp., 54 *Escherichia coli*, 2 *Salmonella enterica*, and 246 *Capnocytophaga* spp. The antimicrobial susceptibility was assessed for the *E. coli* isolates using a panel of 14 antibiotics. The highest level of relative AMR was recorded for ampicillin and sulfamethoxazole. The association found between AMR and the levels of animal welfare scores in shelters was evident although not statistically significant. These results support the hypothesis that the good management of shelters can increase the level of animal welfare, thus reducing the use of antibiotics and, as a consequence, the AMR occurrence found in dogs that share their domestic environment with humans.

## 1. Introduction

Antimicrobial resistance (AMR) is one of the major public health threats that modern society is currently facing and it is one of the biggest recognized priorities facing the World Health Organization [1]. The exposure of microbes to antimicrobial drugs can lead to the development of new AMR mechanisms or to the selection of strains harboring antimicrobial resistance genes and mutations, thus reducing the efficacy of the subsequent therapies [2]. Novel and more effective drug compounds are constantly required to treat infections caused by multi-drug resistant (MDR) strains and prophylactic and therapeutic options that remain available are decreasing at an alarming rate, leading to frequent treatment failures [3,4]. As a consequence of the extensive or inappropriate use of antibiotics, AMR has become a serious threat to both public and animal health worldwide [1,5,6]. National and international public health authorities have launched a response to reduce and mitigate the impact of AMR on a local and on a global scale. Adequate AMR preventive protocols currently in place are based on the systematic surveillance of the prevalence of AMR in bacteria in the food production chain and in humans [7]. In recent years, industrialized countries have implemented their AMR surveillance systems in livestock animals, but little information is actively collected on companion animals [6]. MDR pathogens have been isolated from dogs, demonstrating that companion animals can play a role as a potential reservoir of anti-microbial resistant bacteria that can then be transmitted to humans or other animals. Worryingly, these pathogens can have zoonotic potential [8,9,10,11,12]. Most of the previous research in companion animal reservoirs of AMR has been based on passive surveillance and data collected from hospitalized animals [12,13,14,15,16,17,18,19]. Data on the pathogens isolated from healthy dogs, especially the shelter dog population, is very limited even though these animals have a high potential risk of carrying and spreading a variety of AMR pathogens to both animals and humans [9,14,20]. Moreover, some of the pathogens frequently isolated from both symptomatic and asymptomatic dogs, such as *Pasteurella* spp, *Staphylococcus aureus* (*S. aureus*), *Campylobacter jejuni* (*C. jejuni*), *Salmonella enterica*, and *Escherichia coli* (*E. coli*), due to their zoonotic potential, can pose an important threat for public health [21,22]. A few studies have reported that living with diarrheic pets is a risk factor for campylobacteriosis, salmonellosis, and colibacillosis in humans but the threat to human health in these cases can be recognized and mitigated [22,23,24]. Moreover, dogs that are frequently asymptomatic carriers of *Salmonella enterica* [25] and *Campylobacter* spp. [26] can shed these pathogens in their feces and thus pollute the environment for several weeks, and indeed up to two years [27], for *Campylobacter* spp. and for more than six weeks for *Salmonella enterica*, thus increasing the probability of pathogen transmission [28]. Both pathogens represent a risk factor for humans and in particular, for children, immunosuppressed, and elderly people [29,30]. Direct evidence of *C. jejuni* transmission from a dog to a child living in the same household has been shown [29,31]. Commonly, the most important risk factors for the development of salmonellosis in dogs are related to the consumption of raw meat or poorly cooked meals. Moreover, both probiotics and antibiotics were found to favor carriers of *Salmonella enterica* in dogs [32]. The prevalent species of these pathogens in dogs are *Campylobacter upsaliensis* [27], *S. enterica* serovar *Typhimurium*, and *S. enterica* serovar *Anatum* [25]. Several studies have described the isolation of *E. coli* strains resistant to β-lactam antibiotics and MDR [33] including enteropathogenic strains and carriers of *ast-A* from dogs [34]. Dog housing conditions and shelter infrastructures were identified as risk factors for colibacillosis. Indeed, *E. coli* antibiotic resistance strains have rarely been found in home dogs; instead, in free-roaming dogs and shelter dogs the occurrence of antibiotic resistance strains has been found to be higher [35,36]. Specific factors that contribute to the introduction, persistence, and spread of pathogens in sheltered dogs include high animal population densities, lack of proper veterinary care, stressful and unsanitary housing conditions, limited funding, and high animal turnover [37]. Cultural and socio-economic factors further complicate these problems and put these animals at risk of getting infected, becoming carriers, and transmitting diseases to both animals and humans [38]. Shelter environments in particular can increase the risk of illness because of high animal density, as well as many other factors such as hygiene, food, management, and veterinary interventions which may all play a predisposing role in respiratory and gastrointestinal disease in sheltered dogs. Thus, monitoring for zoonotic pathogens and their AMR in shelter dogs is important for understanding the sanitary risks they pose to the human population and the environment. Therefore, in our study, we aimed to provide data on the occurrence of respiratory and enteric zoonotic pathogens found in a group of 20 dogs housed in each of the 15 shelters located across Italy. We evaluated the antibiotic resistance of *E. coli* strains isolated from the fecal samples with the aim to understand whether environmental conditions and shelter management correlated with the AMR. To reach this objective, the occurrence of AMR in *E. coli* was correlated to sheltered dogs’ welfare scores that were evaluated with the Shelter Quality Protocol (SQP) [39].

## 2. Results

**Figure 1:**
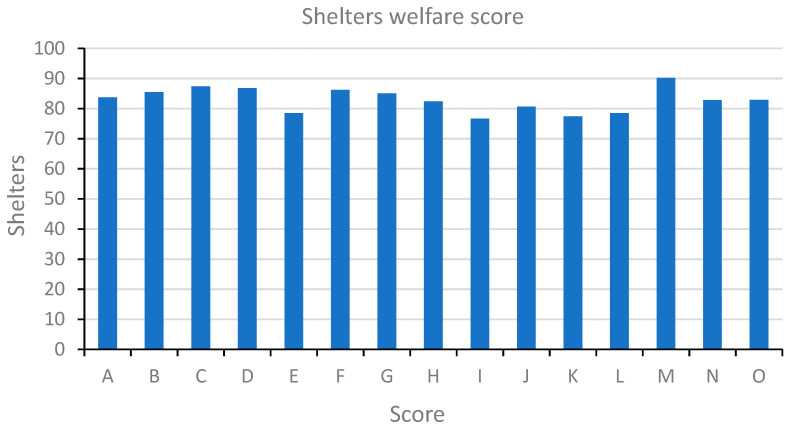
Welfare score of shelters. Graphical representation of shelters welfare scores calculated with the Shelter Quality Protocol. Shelters are identified by capital letters following the same order as Table 1. Shelter welfare score values ranged from 77 to 90 with an average of 83 points.

In this study, we evaluated the welfare of 294 dogs housed in 15 shelters located in 10 Italian regions using a Shelter Quality Protocol (SQP). The average animal welfare score within the shelters was 83 points and ranged from a minimum of 77 points for shelter I to a maximum of 90 points for the municipal shelter M (Figure 1).

In each shelter, nasopharyngeal, rectal, and oral swabs were collected from 20 dogs for a total of 758 swabs to assess the occurrence of the most common zoonotic respiratory and gastroenteric bacteria (Table 1).

A total of 333 samples (43%) were positive in at least 1 of the tests performed, and 7 different bacterial species were detected: *Staphylococcus pseudointermedius* (*S. pseudointermedius*), *Pasteurella multocida* (*P. multocida*), *S. aureus*, *Campylobacter* ssp., *E. coli*, *Salmonella enterica*, and *Capnocytophaga* spp. The bacteria detected in the nasopharyngeal swabs were *S. pseudointermedius* 9/283 (3.1%), *P. multocida* 1/283 (0.35%), and *S. aureus* 9/283 (3.1%). In the rectal swabs, we found *Campylobacter* spp. 12/203 (5.9%), *E. coli* 54/203 (26.6%), and *Salmonella enterica* 1/203 (0.49%). Only shelter H and shelter M tested positive for the *E. coli* O:157 through the detection of presumptive colonies in culture and the subsequent confirmation using PCR identification. In the oral swabs tested by PCR, we detected 246/272 (90.4%) samples positive for *Capnocytophaga* spp. All *Capnocytophaga* spp. isolation attempts from the PCR-positive oral swabs samples failed using our specific agar plates.

**Figure 2:**
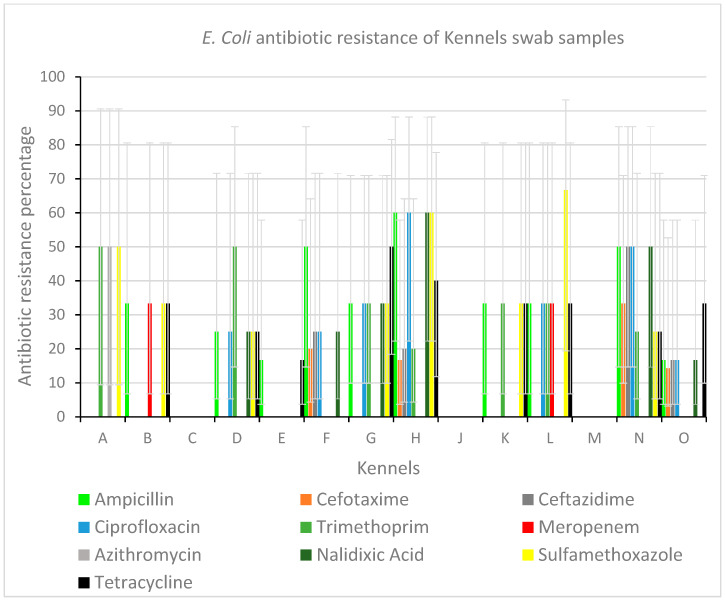
Antibiotic resistance distribution of *E. coli* isolates in sheltered dogs. The histogram represents the relative percentage of antibiotic resistance detected in 14 Italian shelters against 14 compounds that were tested. The relative percentage of antibiotic resistance is shown on the ordinate axis and shelters are listed in capital letters following the same nomenclature indicated in Table 1. In shelter I, no data were collected.

We assessed AMR in *E. coli* strains isolated from dogs housed in 14 shelters using a panel of 14 antimicrobial agents. We found that the AMR profiles of *E. coli* isolates differed for each sampled shelter when considering all the compounds tested but, overall, resistance to ampicillin was detected most frequently (Figure 2). The highest number of strains resistant to at least one class of antibiotics was found in shelter L where 66% of strains were resistant to sulfamethoxazole. Moreover, resistance to one or more antibiotics was detected in at least 50% of *E. coli* isolated in 6 shelters (A, D, F, H, L, and N). Resistance to the third-generation cephalosporins was observed in strains isolated in four shelters only, including shelter N, where we detected 50% of strains resistant to these antimicrobials. All tested isolates were susceptible to gentamicin, tigecycline, chloramphenicol, and colistin. Shelter A was the only one where *E. coli* isolates showed resistance to azithromycin. Interestingly, isolates from three shelters, C, J, and M, were fully susceptible to all the tested antimicrobials (Figure 2).

**Figure 3:**
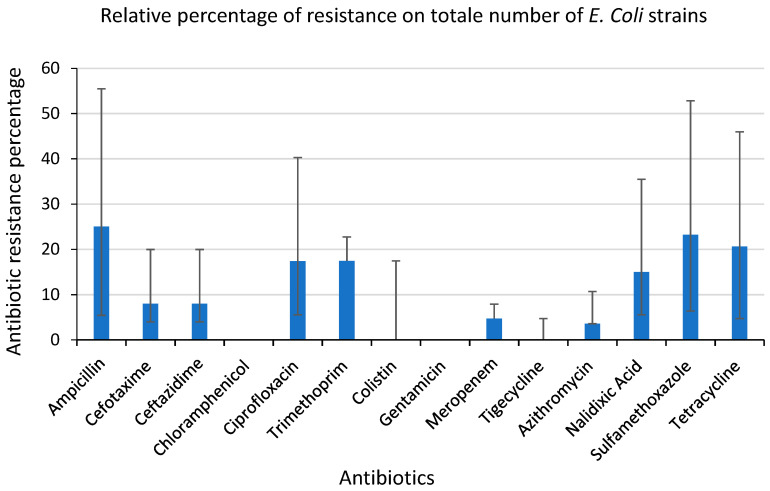
Distribution of antibiotic resistance of the total *E. coli* isolates in the 14 shelters. The histogram represents the relative percentage of antibiotic resistance detected on the total number of *E. coli* isolates against the 14 compounds that were tested. The relative percentage of antibiotic resistance is shown on the ordinate axis and antibiotics on the abscissa axis.

The overall occurrence of resistance to each antimicrobial was ampicillin 25.3%, sulfamethoxazole 23.21% tetracycline 20.61%, trimethoprim 17.45%, ciprofloxacin 17.36%, nalidixic acid 14.98%, cefotaxime and ceftazidime 7.98%, meropenem 4.71%, and azithromycin 3.57% (Figure 3).

*E. coli* samples were tested for multidrug resistance (MDR), which was found in a total of 14 strains and showed a complex multidrug resistance pattern (Table 2).

MDR isolates showed 13 different phenotypes and were detected in 10 shelters: one strain in shelters A, B, D, F, K, L, and O; two strains in shelters G and N; and three strains in shelter H. Interestingly, we found the same pattern of antibiotic resistance in shelter D, G, and H. In shelter H, we found a total of three MDR strains of which one was resistant to four antibiotics, one to five antibiotics, and the last strain was resistant to six compounds (Table 2).

The shelters were divided into two groups based on the values of welfare score recorded with the SQP. Each shelter was classified into a high- or low-level category if its SQP score value was greater or lower than the median welfare scores value of 83.42 calculated, respectively. Thus, shelters H, L, J, K, N, O, and E were included in the low category while shelters D, B, C, A, F, M, and G were in the high category. The differences between the high- and low-level categories were statistically significant (*p* value < 0.001).

**Figure 4:**
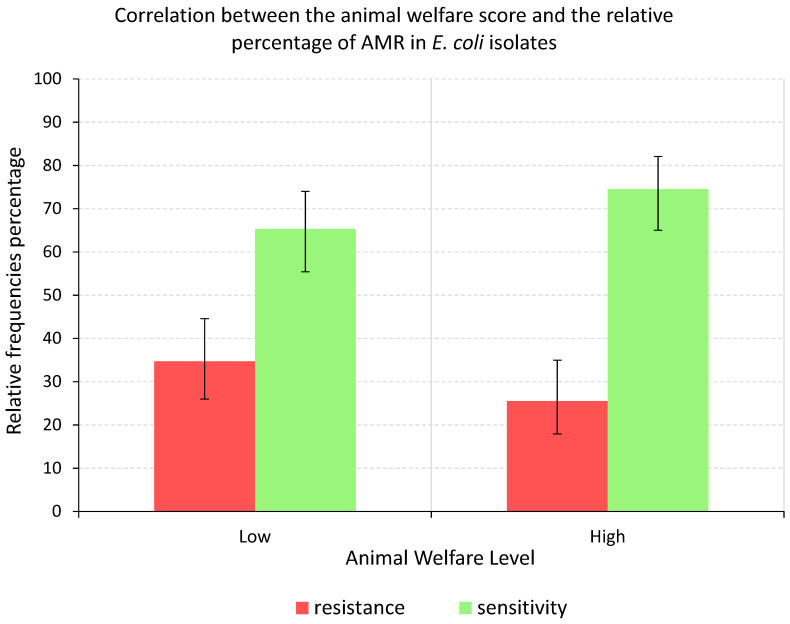
Graphical representation of the correlation between the animal welfare score and the relative percentage of AMR in *E. coli* isolates. The graph is divided into two parts based on the grouping of the shelters in high and low welfare scores, each including two columns: one green and the other red. The green columns represent the frequencies of antibiotic-sensitive strains while the red columns represent the frequencies of antibiotic resistance. Error bars represent the levels of confidence of the percentage values observed. These ranges were calculated with a Bayesian approach using the Beta distribution.

To evaluate the possible correlation between the two shelter categories (Low and High) and the antibiotic resistance or sensitivity level recorded (Res. and Sen.), two statistical tests were performed. First, the chi-square test was used to identify the possible differences between the two (Low and High) shelters welfare score groups and the resistance/sensitivity to antibiotics found in the 14 shelters sampled (Figure 4). The shelters classified in the category of a low-level welfare score showed an AMR frequency of 35%, while the shelters belonging to the category of a high-level welfare score showed an AMR frequency of 26%. On the other hand, the shelters with a high level welfare score showed a high number of antibiotic-sensitive strains (frequency of 74%) while shelters belonging to the low level welfare score category showed a lower level of antibiotic-sensitive strains (frequency of 65%) (Figure 4).

However, the observed differences between the dog welfare scores and the frequencies of resistance/sensitivity of *E. coli* strains were not significant (*p* = 0.465). For the second statistical test, we adopted a Bayesian approach and the Low and High welfare score categories overlapped with the Beta distribution curve of the AMR values found in the samples. Therefore, no statistically significant differences were identified between the animal well-being score and the AMR frequencies recorded.

## 3. Discussion

In this study, we aimed to collect data on the occurrence of respiratory and enteric zoonotic pathogens found in sheltered dogs across Italy. We additionally evaluated the antimicrobial resistance of *E. coli* strains isolated from the dogs to understand whether the AMR levels correlated with shelter management and the dogs’ welfare [40]. To reach our final goal, we measured the welfare score of sheltered dogs according to the Shelter Quality Protocol which allowed us to have an overall assessment of the management, environment, and animal welfare. Using a scale from 1 to 100 points, the SQP score achieved in the 15 shelters sampled ranged from 77.4 to 90 points. Thus, SQP scores indicated the overall good management of the tested shelters. Interestingly, the highest number of respiratory pathogens was isolated in shelter E which was classified as having a low SQP score; moreover, it was the only place where we isolated *P. multocida.* Additionally, L, H, and N shelters, belonging to the low SQP score shelters group, showed the highest relative percentages of AMR for sulfamethoxazole, ciprofloxacin, and cephalosporins. Overall, the AMR levels observed for these antimicrobials in sheltered dogs were low when compared with reported data in livestock in Italy [41]. It is well known that the use of broad-spectrum antibiotics without proper veterinary assessment can lead to the development of AMR through mechanisms and the selection for AMR strains [42]. In small animal clinical practice, fluroquinolones and cephalosporins are the most used antibiotics [33] together with other β-lactams [10] such as amoxicillin and ampicillin [43]. Indeed, in our samples, ampicillin showed the highest AMR average value among all compounds tested. Moreover, the relative percentage values of AMR against cephalosporins we detected in *E. coli* isolates were higher than those found in livestock that ranging from 0.2% up to 1.1% [41,42,43,44]. In addition, our data showed the presence of several MDR strains with combined resistance to fluoroquinolones and cephalosporins while this combination is usually rare to be found in food production animals that ranged from 0% up to 0.5% [41].

The samples collected from the dogs housed in the monitored shelters allowed us to detect the main respiratory and gastroenteric zoonotic pathogens. The data we obtained showed a lower occurrence of main zoonotic pathogens including *Campylobacter* spp, *Salmonella* spp., and *P multocida* when compared to the bacteria occurrence found in the literature for domestic dogs, thus indirectly suggesting the good management of the structures examined [45,46,47]. Among bacterial agents detected, we found frequent colonization of the oral cavity by *Capnocytophaga* spp. and in particular *C. canimorsus* and *C. cynodegmi* that are commensal microbes thriving in the oral cavities of dogs. *C. canimorsus* can cause a fatal systemic infection in immunocompromised humans [48]. The isolation and identification of bacteria from the samples was difficult because of the long incubation times required for the bacterium to grow [49]. The high percentage of positive oral swabs we recorded was probably a consequence of the high PCR test sensitivity when compared to the isolation procedure. Indeed, the molecular techniques are the gold standard for bacterial identification of *Capnocytophaga* spp. [50].

Within all the bacteria isolated from our samples, we choose to assess AMR in *E. coli* as it is considered a representative indicator of antimicrobial resistance and the most common Gram-negative bacterium present in animal feces [36]. Moreover, *E. coli* readily acquires conjugative plasmids carrying AMR genes that can then be transferred to other Enterobacteriaceae in the gut microbiome of animals and humans; thus, it is potentially able to transfer the AMR phenotype [36]. We detected a total of 54 *E. coli* strains showing resistance to 1 or more of the 14 antimicrobials tested and we found that the most common resistances were against ampicillin, confirming the data obtained in other studies for the latter compound [36,42]. In our work, 14 *E. coli* strains showed the MDR phenotype which represented 26% of the total strains isolated. This percentage was comparable to the MDR data found in dogs from the USA where the detected MDR percentage in dogs reached 28.9% [51] while it was lower than the MDR described in dogs in Japan (43%) [52] and the 66.8% detected in Poland [33]. Two MDR strains were resistant to meropenem that belongs to the carbapenem class. Several epidemiological studies have recently revealed the emergence of MDR bacterial pathogens worldwide, thus posing a new public health threat [53,54].

Interestingly, we show a tendency that suggests an association between the shelter’s welfare score and the presence of *E. coli* AMR strains. Furthermore, the highest number of MDR strains was found in N and H shelters which were also the shelters classified in the lower welfare score group. In order to correlate the AMR frequencies found in *E. coli* strains with the SQP score, shelters were divided into two groups. Each shelter was classified into a high- or low-level category when its welfare score value was, respectively, greater or lower than the shelter’s welfare scores median value. Shelters with high SQP scores, in general, showed the tendency to correlate with a lower percentage of AMR, and vice versa, lower SQP displayed the tendency to correlate with higher AMR values. Although this association did not prove to be statistically significant, we believe that the measurement of antibiotic resistance frequencies can be an indirect way to evaluate animal welfare in shelters, as it might be indicative of the selective pressure exerted by the antibiotic therapies that the animals received.

The total number of samples collected over in 10 out of 20 Italian regions makes this study representative of a large sheltered dog population. Moreover, to our knowledge this is the first time a correlation between sheltered dogs’ well-being and AMR was studied. We found an association between animal welfare score and AMR, but we did not prove a statistical significance for the data shown, possibly due to two significant limitations that affected our results. Firstly, the shelters were recruited on a voluntary basis which constitutes a bias as likely, only shelters with good management participated in the study. This hypothesis was confirmed by the high SPQ score registered by all shelters; as a consequence, we were missing samples from shelters with a very low management score. Secondly, given the low differences in the SPQ scores registered between the two categories of shelters (high and low), the number of samples collected was not representative enough. Despite these two limitations, and considering that these were the first available data on the presence of AMR profiles in shelters where animal welfare has been assessed, for the first time we identified a tendency that could link AMR percentage and sheltered dogs’ welfare scores. Although, further studies are needed to confirm our data, the work presented here could help to better understand the dynamics of antimicrobial resistance in animals that closely share the domestic environment with humans.

## 4. Materials and Methods

### 4.1. Animal Welfare

The welfare assessment of dogs was performed by applying the Shelter Quality Protocol (SQP) that was been published by De Massis et al. [55]. Briefly, the SQP was developed following the criteria of reliability, validity, and feasibility [39,40] in order to assess the overall welfare level of shelter dogs using a mobile device application “Shelter version 1.2.4-SNAPSHOT” produced by Istituto Zooprofilattico Sperimentale Abruzzo e Molise “G. Caporale” (IZSAM, Teramo, Italy) [38,56,57,58,59,60,61,62,63,64,65,66,67,68,69,70].

Since welfare is the outcome of multifactorial effects influencing animals’ life, several variables were considered when applying the SQP. The protocol was conducted via direct observation of the animals in response to environmental stimuli and identified important aspects of the shelter management by identifying the presence of welfare hazards [39,56]. The criteria selected to evaluate animal welfare at the shelter level included management procedures (management-based measures), the housing environment (resource-based measures), and direct welfare outcomes (animal-based measures). The evaluation of animal welfare according to the SQP was carried out using the data collected at three different levels: measures taken at the shelter level; measures taken at the pen level, which were selected randomly within the shelter; and measures taken at the animal level. These three measures were based on criteria that include different principles. We used a Delphi procedure based on the assumption that the final result of the analysis can be considered reliable only when the group of expert examinators has established an agreement on each criterion adopted in order to obtain a final “welfare score”. Furthermore, the operator assigning a score to a specific categorical variable had to answer a set of different questions that were associated with each different welfare criteria using a specific scale of values (for example the body condition score measure can be assessed as “adequate, too thin or too fat”). The animal welfare experts established the relative weights for each criterion using a scale from 0 to 100. The answers to all the questions contained in the protocol were then weighted to obtain the shelter score value expressed as a percentage of the “ideal shelter index value” which was set to 100. A well-being score was assigned to each shelter based on the values obtained in the randomly selected dogs housed in the facility. The scoring system allows us to measure how much the overall level of animal welfare in a given shelter differs from the “ideal animal shelter welfare”. For example, a shelter that reaches an 80 points index differs 20 points from the “ideal shelter” score [55].

The scoring system is based on a binary evaluation system that derives from the relationship between positive and negative indicators. The Delphi procedure was used to measure each criterion and obtain the “welfare score” for each animal. The sum of all the measures taken by assessing the animals was summarized to obtain the shelter total score and the final score of all shelters was subsequently compared by the app with the ideal shelter value that was arbitrarily fixed at 100 [39,40,41,42,43,44,45,46,47,48,49,50,51,52,53,54,55].

### 4.2. Animals and Samples

The participation of shelters in this study was on a voluntary basis. The study included dogs housed in 15 shelters covering 10 Italian regions (Lombardy, Piedmont, Emilia Romagna, Marche, Abruzzo, Lazio, Campania, Puglia, Basilicata, and Calabria). SQP was applied to a maximum number of 20 boxes randomly selected in each shelter. Only animals with more than six months of age and housed for at least two months in a given shelter were considered. Dogs sampling was performed as previously described [55].

The sample size was calculated on the basis of the work of Dell et al. (2002) for dichotomous variables. In order to highlight a minimum (statistically significant) difference of 80% between the proportions of each pair of groups compared, a confidence level of 95% and a test power of 80% were chosen which determined a minimum sample size of 20 animals [57]. This sample size was chosen based on the prediction of having 1% pathogen prevalence with a 95% confidence level. The study enrolled 294 dogs and each dog was sampled with nasopharyngeal, rectal, and oral swabs. Rectal swabs were not sampled in shelter I for an unforeseen logistic problem. Samples were transported to the laboratory in refrigerated conditions (4 °C) in Amies agar medium (Liofilchem S.r.l., Teramo, Italy) and processed within 48 h after collection.

### 4.3. Bacterial Isolation and Identification

Bacteria were isolated by plating the swabs onto three different media: 5% blood agar, Gassner agar (Liofilchem S.r.l., Teramo, Italy) and mannitol salt agar (Liofilchem S.r.l., Teramo, Italy). Plates were incubated at 37 °C in aerobic conditions for 72 h. The colonies were screened by morphology and β-hemolysis ability on blood agar. Biochemical identification was performed using an automated microbial detection system (Vitek 2 Compact, BioMèrieux, Marcy-l’Étoile, France) according to the manufacturer’s recommendation.

Starting from an aliquot of each rectal swab, the isolation of *Campylobacter* spp. was performed according to EN ISO 10272 1:2017 [58]. Briefly, *Campylobacter* spp. isolated strains were subcultured on Columbia blood agar plates in microaerophilic conditions at 42 °C for 48 h. The DNA was extracted from the colonies by Maxwell^®^, an automated DNA extraction system (Promega, Milano, Italy), and quantified using a spectrophotometer (Nanodrop Products, ThermoScientific, Wilmington, DE, USA). Colonies were confirmed as thermotolerant *Campylobacter* spp. using molecular identification PCR, as previously described [59,60].

The isolation of *Salmonella enterica* was carried out with an internal procedure accredited in accordance with international standard methods (ISO 6579-1:2017) [61]. Briefly, rectal swabs were pre-enriched in buffered peptone water by incubating at 37 °C for 24 h. The pre-enriched suspension was tested on Rappaport-Vassiliadis semi-solid modified medium (IZSAM, Teramo, Italy) and Muller-Kauffmann tetrathionate novobiocin medium (IZSAM, Teramo, Italy) and incubated at 37 °C for 24 h. After visual selection, colonies were placed on two selective agar media: Rambach agar and Xylose-Lysine-Deoxycholate agar (Microbiol Diagnostics, Cagliari, Italy). Finally, the colonies were collected and inoculated on triple sugar iron agar and incubated at 37 °C for 24 h. Colonies were confirmed as *Salmonella enterica* based on their ability to ferment glucose and produce H_2_S. Colonies referable to *Salmonella enterica* were confirmed by an automated microbial detection system (Vitek 2 Compact, bioMérieux, Marcy-l’Étoile, France) and subsequently identified by agglutination with specific antisera (Statens Serum Institut, Copenhagen, Denmark).

Two agar media were homemade for the detection of *Capnocytophaga* spp. in culture plates starting from the oral swabs. The first medium was VCAT [62] and consisted of a Columbia 10% Cooked Horse Blood Agar supplemented with Vancomycin/Colistin-sulphate/Amphotericin/Trimethoprim (VCAT supplement, Oxoid, Rodano, Italy) and a multivitamin supplement complex (Vitox, Oxoid). The second medium was the modified Thayer–Martin and was produced according to Bargery’s protocol [63] and consisted of a GC agar (GC agar medium, Oxoid) supplemented with Colistin methasulfonate/Nystatin/Vancomycin (VCN selective supplement, Oxoid) and a multivitamin complex (Vitox, Oxoid, Rodano, Italy). Samples were seeded on both culture media and incubated at 37 °C for 48 h in controlled atmosphere jars with 5% CO_2_ (AnaeroGen™, Thermo Scientific, Monza, Italy). Any colonies with a morphology of short, rod shaped, or long stranded Gram-negative cells were isolated and reinoculated on blood agar plated under the same conditions, as previously described. Colonies with a “gliding movement” aspect on the agar surface were selected for being identified with a biomolecular method.

### 4.4. Detection of Capnocytophaga Canimorsus/Cynodegmi

From each oral swab, *Capnocytophaga canimorsus* and *C. cynodegmi* were detected by PCR as described by Suzuki et al. [50]. *C*. *canimorsus* NCTC 11,921 and *C. cynodegmi* NCTC 12,243 strains were used as positive controls. DNA was extracted from swabs and control strains using the Maxwell 16 Cells and Tissue DNA purification Kit (Promega, Milano, Italy). Partial 16S rRNA gene fragment amplification products were identified through QIAxcel equipment using the recommended standard protocol (Qiagen, Hilden, Germany).

### 4.5. Detection of E. coli O:157 in Rectal Swabs Samples

The detection of *E. coli* O:157 was carried out with an internal procedure according to the WOAH Manual of Diagnostic Tests and Vaccines for Terrestrial animals [64]. The fecal material collected by swabs was inoculated on two selective media: Tellurite cefixime sorbitol Mac Conkey Agar (CT-SMAC, IZSAM, Teramo, Italy) and Mac Conkey Sorbitol Agar (SMAC, IZSAM, Teramo, Italy). The same swabs were then inoculated in 10 mL broth enriched with Novobiocin, Modified Tryptone Soya Broth (mTB, IZSAM, Teramo, Italy). The selective agar media and the broth were incubated at a temperature of 37 °C for 18–24 h and the suspect colonies were detected on selective mediums and re-isolated on Nutrient Agar (IZSAM, Teramo, Italy). After 18–24 h, each broth culture was subsequently tested with immunomagnetic particles binding anti-O157 antibodies (Dynabeads anti *E. coli* O157, Thermofisher Scientific Baltics, UAB, Vilnius, Lithuania) through a magnetic shaker (Dynabeads MX Mixer, Invitrogen, Waltham, MA, USA). In brief, after the magnet was activated, the supernatant was eliminated and the cells immunocaptured by the magnet were resuspended in a pH-neutral physiological solution. Finally, the suspension was seeded with the same procedure applied to the initial material. The typical colonies detected were identified through the agglutination test using a specific antiserum against *E. coli* O:157 (Wellcolex *E. coli* O:157, Remel Europe Ltd., Lenexa, KS, USA) and with biochemical (Vitek) and/or biomolecular identification [64].

### 4.6. Characterization of E. coli Isolates

*E. coli* strains isolated from rectal swabs were biochemically identified using standard biochemical methods and subsequently tested by PCR to detect the *eae* gene which encodes intimin (an adesin) and the typical of Verotoxigenic *E. coli* (VTEC) genes of virulence (*vtx1*, *vtxe*, *vtx2f2*), using an *E. coli* O:157 strain positive for *eae, vtx1*, and *vtx2* and a strain of *E. coli* O28 strain positive for *vtx2f*-positive as positive controls with both being supplied by EU-RL-ISS. Deoxyribonucleic acid (DNA) extraction from the samples was carried out using the Maxwell instrument through the Maxwell 16 Cells and Tissue DNA Purification Kit (Promega, Milano, Italia) as well as the PCR test which was performed using Master Mix (Qiagen, Milano, Italia) following the standard protocol. The sets of primers (Eurofins Genomics, Milano, Italia) used for each *E. coli* gene were as follows eaeAFw 5′-GACCCGGCACAAGCATAAGC-3′, eaeARw 5′-CCACCTGCAGCAACAAGAGG-3′, stx1Fw 5′-ATAAATCGCCATTCGTTGACTAC-3′, stx1Rw 5′-AGAACGCCCACTGAGATCATC-3′, stx2Fw 5′-GGCACTGTCTGAAACTGCTCC-3′, stx2Rw 5′-TCGCCAGTTATCTGACATTCTG-3′, 128-1Fw 5′-AGATTGGGCGTCATTCACTGGTTG-3′, and 128-2Rw 5′-TACTTTAATGGCCGCCCTGTCTCC-3′. The samples were amplified using the thermocycler ProFlex PCR System (Applied Biosystems, Monza, Italia) with an initial denaturation at 95 °C for 5 min, followed by 35 cycles of amplification with a denaturation phase at 95 °C for 1 min, an annealing at 65 °C for 1 min, and a final extension at 72 °C for 1.5 min. The PCR test results were visualized using the QIAxcel instrument and analyzed with the QIAxcel Screen gel Software (Qiagen, Milano, Italia).

### 4.7. Antimicrobial Susceptibility

*E. coli* isolated strains were characterized phenotypically by broth microdilution using Sensititre^®^ EUVSEC^®^ plates (Thermofisher Scientific, Paisley, UK). The antibiotics on the EUVSEC^®^ plate were ampicillin (1–64 µg/mL) (AMP), azithromycin (2–64 µg/mL) (AZY), gentamicin (0.5–32 µg/mL), tigecycline (0.25–8 µg/mL) (TGC), ceftazidime (0.5–8 µg/mL) (CAZ), cefotaxime (0.25–4 µg/mL) (CTX), colistin (1–16 µg/mL) (CST), nalidixic acid (4–128 µg/mL) (NAL), tetracycline (2–64 µg/mL) (TET), trimethoprim (0.25–32 µg/mL) (TMP), sulfamethoxazole (8–1024 µg/mL) (SUL), chloramphenicol (8–128 µg/mL) (CHL), meropenem (0.03–16 µg/mL) (MEM), and ciprofloxacin (0.015–8 µg/mL) (CIP). The quality control of the batch was performed with *E. coli* ATCC^®^ n. 25922. The antimicrobial test was carried out on colonies grown on non-selective agar medium (Tryptic Soy Agar, VWR, Milan, Italy) incubated at 37 °C overnight, subsequently touched with a sterile swab, and transferred into sterile vials with sensitive demineralized water using a sterile swab (Thermofisher Scientific, Paisley, UK) until samples reached 0.5 McFarland turbidity. Then, 0.1 mL of each suspension was inoculated into cation-adjusted Mueller Hinton Broth (Thermofisher Scientific, Paisley, UK) and 50 µL of this suspension was added into the wells of the EUVSEC^®^ plates. The plates were incubated aerobically at 37 °C for 18 h before the reading. The definition of sensitivity or resistance was based on the Epidemiological Cut Off value (ECOFF) for all antibiotics except azithromycin, for which CLSI M100 was used [65,66]. The strains were referred to as multi-drug resistant (MDR) when they simultaneously showed resistance to at least three different classes of antimicrobial drugs.

### 4.8. Statistics Analysis

Each shelter was evaluated considering all 14 antibiotics tested. If antimicrobial resistance to at least one of the 14 antibiotics was found, the shelter was included in the antibiotic-resistant group. The 14 shelters were divided into two categories (low score and high score) depending on whether the well-being SQP score value recorded was lower or higher than their median value, respectively. To test the differences in the animal welfare scores of the two shelter groups, a Mann–Whitney test was applied.

A chi-square test was then applied to check for differences between the well-being category (low–high) and the presence or absence of antibiotic resistance in the samples tested [67]. The statistical analysis was carried out using the XlStat Premium 2022.2.1 Addinsoft software (XLStat, Paris, France) [68]. Moreover, using a Bayesian approach a 95% confidence interval (95% CI) of the antibiotic resistance percentage in the two shelter groups (high and low welfare) was calculated by applying a Beta distribution and the categories were compared [69].

## 5. Conclusions

The data collected in our study highlights how sheltered dogs can be a potential reservoir of zoonotic pathogens and of AMR in *E. coli* strains. These data suggest that there is a health risk factor that needs to be taken into consideration when designing protocols of public health measures on AMR because humans share the same environment with dogs. Although not statistically significant, we show a tendency that suggests an association between animal welfare and the presence of AMR in bacterial strains, thus good management of shelters can increase animal welfare, reduce the use of antibiotics, prevent AMR, and reduce human exposure in a “one health” perspective. Moreover, our data suggest that SQP is a useful tool to measure animal welfare in sheltered dogs and the protocol could be adopted by the Veterinary Services as an official assessment tool in shelters.

Future research on the animal–human interface will help formulate and implement preventive measures against the increase in antimicrobial resistance and, in this context, dogs could be instrumental in helping to monitor the health conditions in the domestic environment.

## Figures and Tables

**Table 1:** Zoonotic bacteria detected in Italian shelters. The main agents of zoonotic interest detected in dogs are grouped by shelter site and swab type. Capital letters identify shelters and pathogens detected on the nasopharyngeal, rectal, and oral swabs, are listed. The row at the bottom of the table shows the total number of swabs performed and the total number of detected pathogens.

	Nasopharyngeal Swabs				Rectal Swabs				Oral Swabs	
Shelters	Total n.	*S. pseudointermedius*	*P. multocida*	*S. aureus*	Total n.	*Campylobacter* spp.	*E. coli*	*Salmonella* enterica	Total n.	*Capnocytophaga* spp.
**A**	20	0	0	2	17	1	2	0	20	14
**B**	18	0	0	0	10	1	3	0	19	14
**C**	17	0	0	1	14	0	1	0	18	14
**D**	16	0	0	0	16	0	4	0	15	14
**E**	20	5	1	1	20	2	6	0	20	20
**F**	20	1	0	0	11	0	4	1	20	20
**G**	18	0	0	0	16	0	6	0	20	20
**H**	20	0	0	0	9	0	5	0	20	18
**I**	18	0	0	2	15	3	ND ^1^	0	20	20
**J**	20	0	0	1	16	0	2	0	20	17
**K**	20	0	0	0	8	0	3	0	20	18
**L**	19	0	0	0	12	2	3	0	20	17
**M**	20	1	0	1	13	3	5	0	ND ^1^	ND ^1^
**N**	19	2	0	1	11	0	4	0	20	20
**O**	18	0	0	1	15	0	6	0	20	20
**Total**	**283**	**9**	**1**	**9**	**203**	**12**	**54**	**2**	**272**	**246**

^1^ ND = not determined **A.** Forlivese district shelter (Forli-Cesena); **B.** Tre Ponti municipal shelter of Fano (Pesaro-Urbino); **C.** Municipal shelter of Ravenna (Ravenna); **D.** LIDA Ortona municipal shelter (Chieti); **E.** Varca group shelter Cassano allo Ionio (Cosenza); **F.** Montesilvano Dog Village (Pescara); **G.** Shelter refuge ENPA Novara (Novara); **H.** Shelter refuge Recchia (Frosinone); **I.** Ottaviano Dog Park (Napoli); **J.** Cynophilist center of Taburno (Benevento); **K.** National dog league Ruvo di Puglia (Bari); **L.** Lucania dog s.r.l (Potenza).; **M.** Intermunicipal shelter–cattery Park (Monza e Brianza); **N.** Municipal refuge of Molfetta (Bari); **O.** Raffo shelter refuge (Varese).

**Table 2:** List of MDR *E. coli* strains detected in the 14 shelters differentiated by shelter and group of drugs. The *E. coli* strains that showed resistance to at least three antibiotic compounds were considered MDR. Each MDR *E. coli* isolate is expressed as a percentage of the total number of strains.

Shelters	MDR	Total	Percentage Number of MDR Strains
A	TMP-AZY-SUL ^1^	1	1.85
B	AMP-MEM-SUL-TET	1	1.85
D	AMP-CIP-TMP-NAL-SUL-TET	1	1.85
F	AMP-CTX-CAZ-CIP-NAL	1	1.85
G	AMP-CIP-TMP-NAL-SUL-TET	2	3.7
H	AMP-CIP-NAL-SUL	1	1.85
H	AMP-CIP-TMP-NAL-SUL-TET	1	1.85
H	AMP-CIP-NAL-SUL-TET	1	1.85
K	AMP-TMP-SUL-TET	1	1.85
L	AMP-CIP-TMP-SUL-TET	1	1.85
N	AMP-CTX-CAZ-CIP-TMP-NAL-SUL-TET	1	1.85
N	AMP-CTX-CAZ-CIP-NAL	1	1.85
O	AMP-CTX-CAZ-CIP-NAL-TET	1	1.85
	NO MDR	40	74.1

^1^ AMP. ampicillin; AZY. azithromycin; CAZ. ceftazidime; CHL. chloramphenicol; CIP. ciprofloxacin; CTX. cefotaxime; MEM. Meropenem; NAL. acid nalidixic; SUL. sulfamethoxazole; TET. tetracycline; TMP. Trimethoprim.

## Data Availability

Data analyses and protocols will be available upon request.
